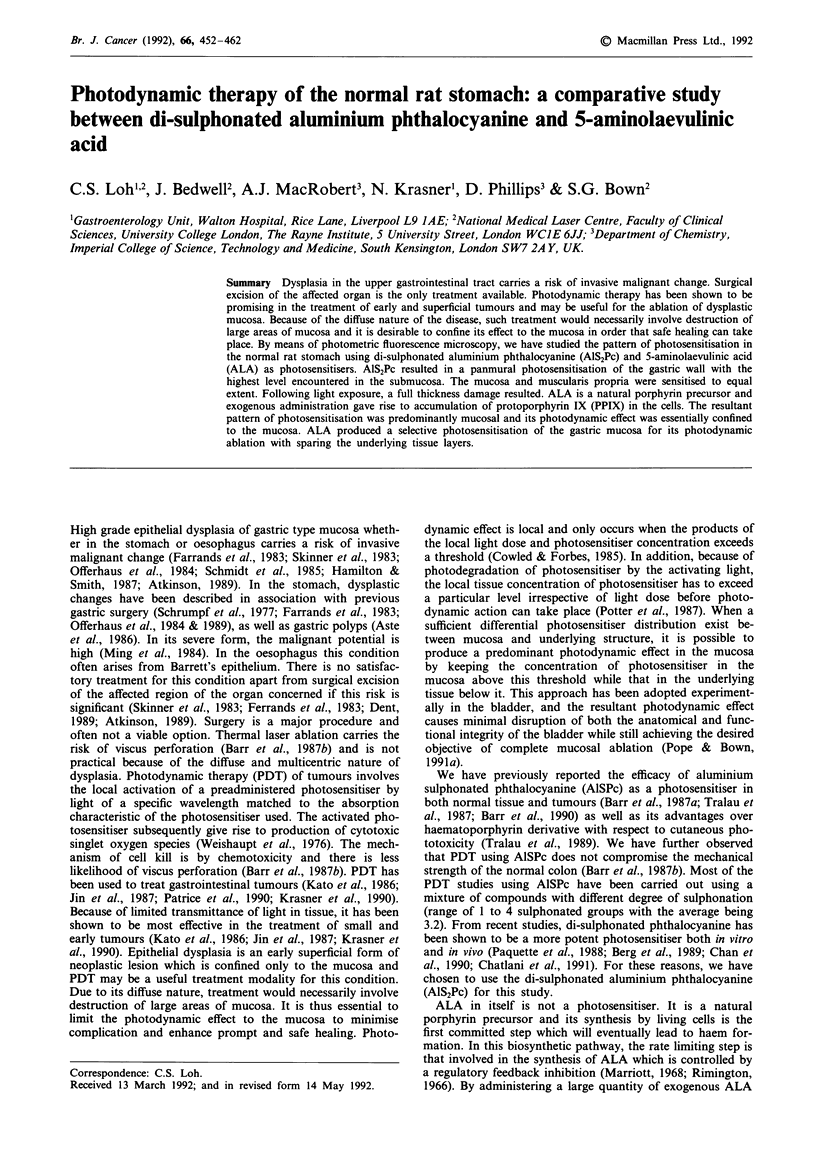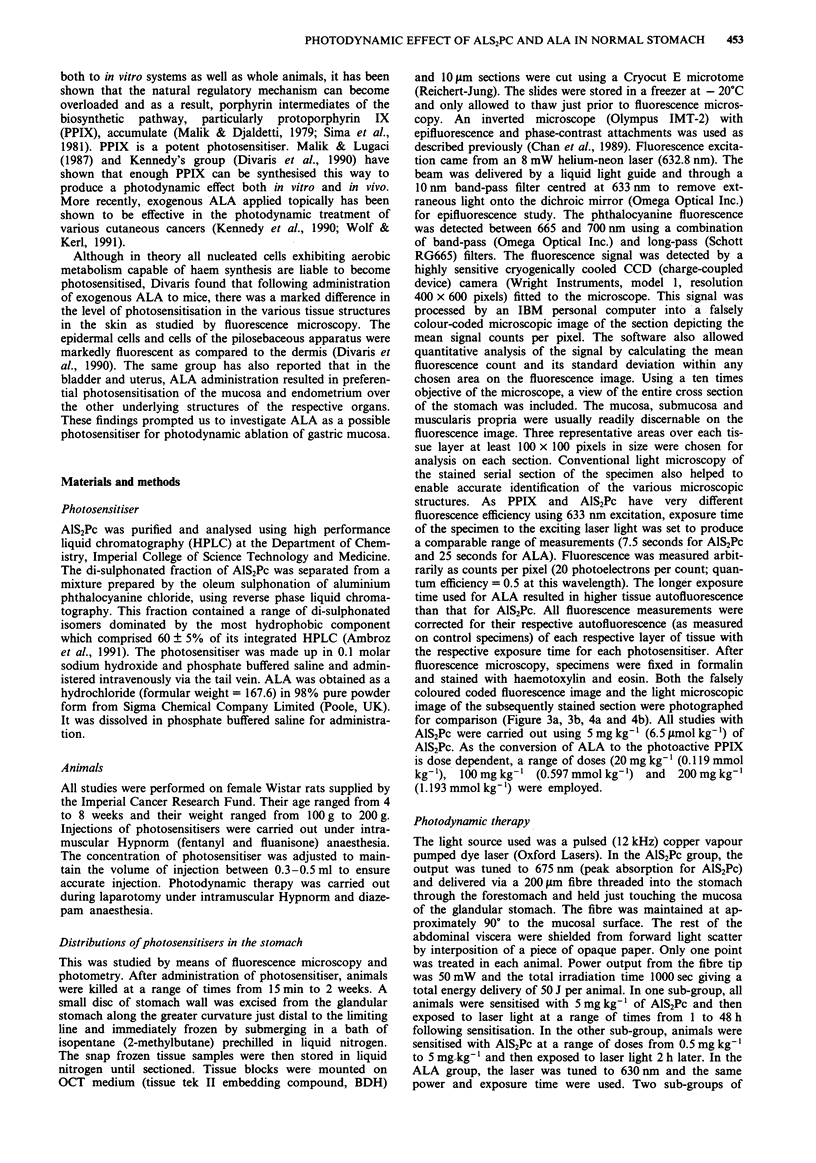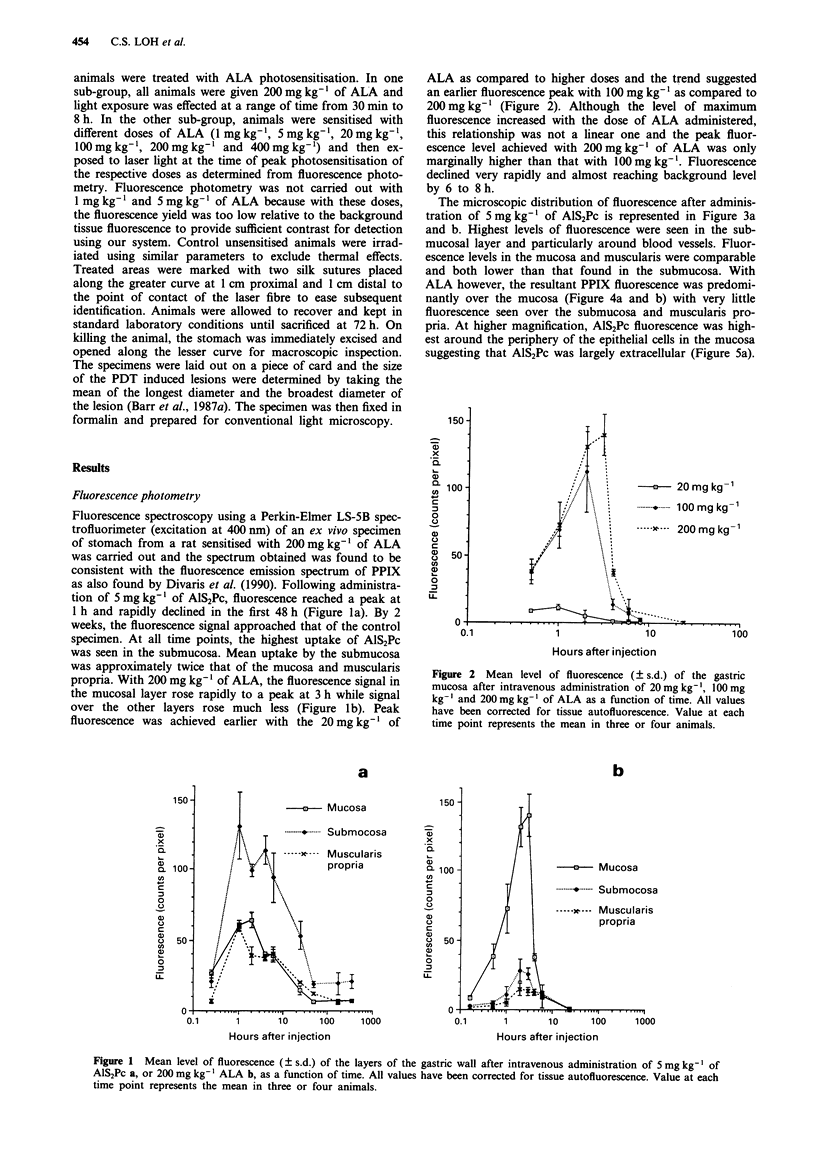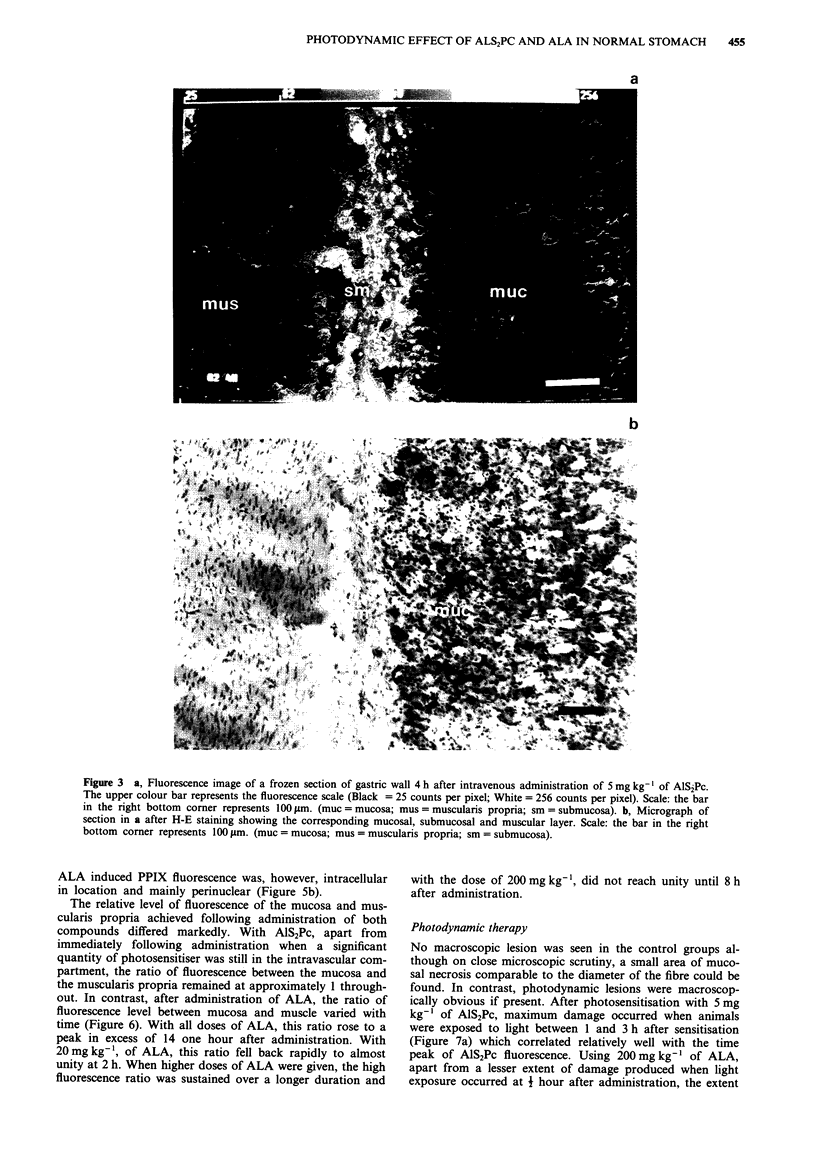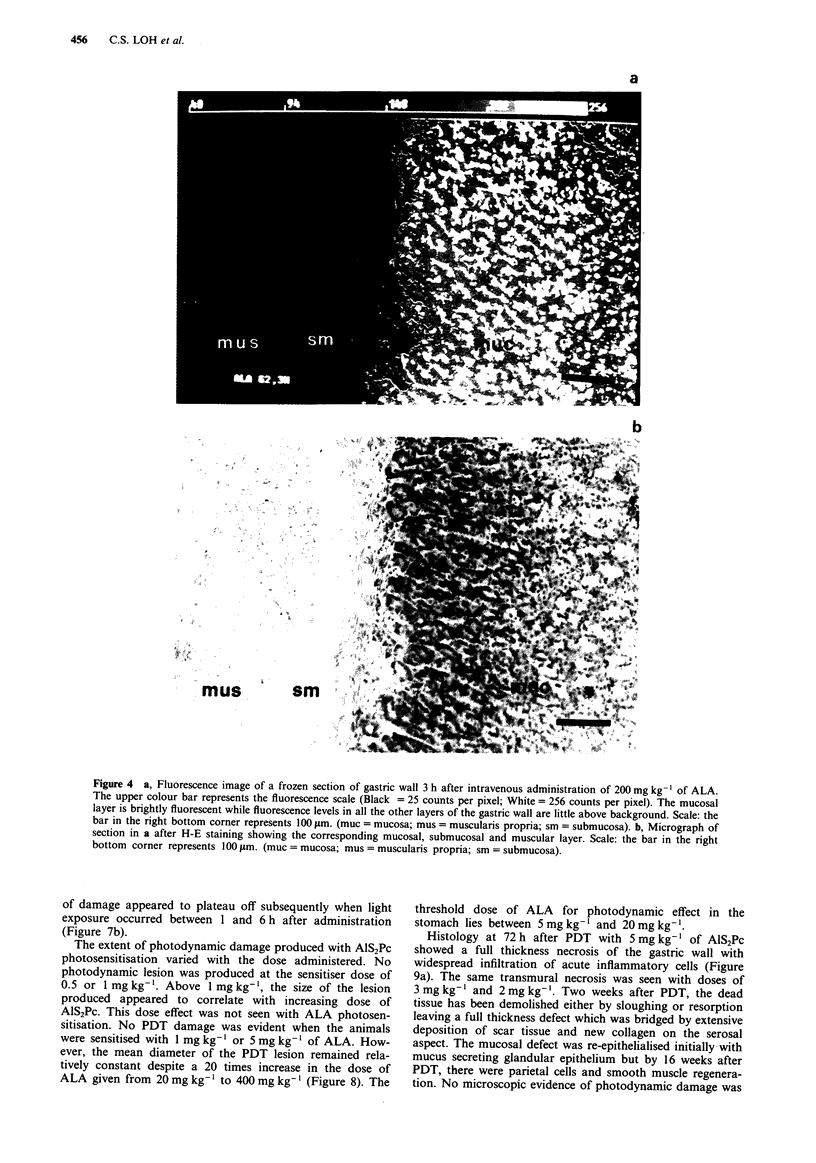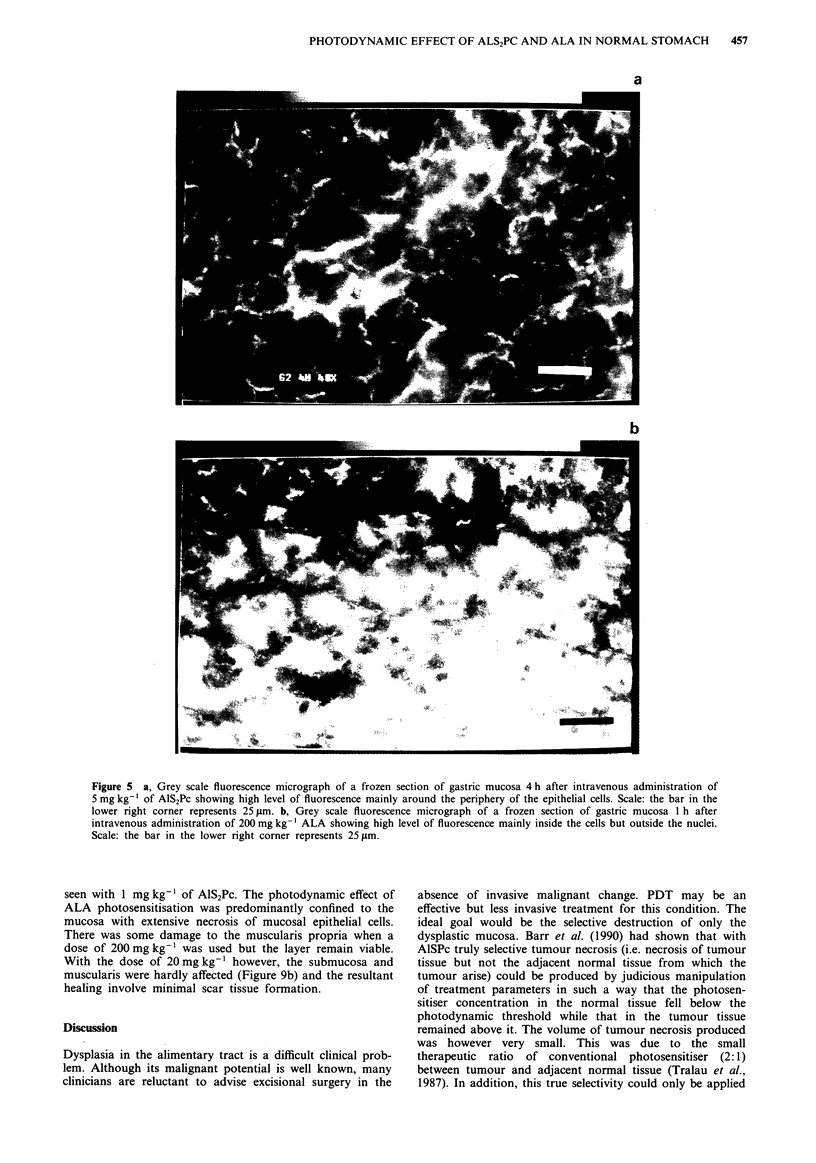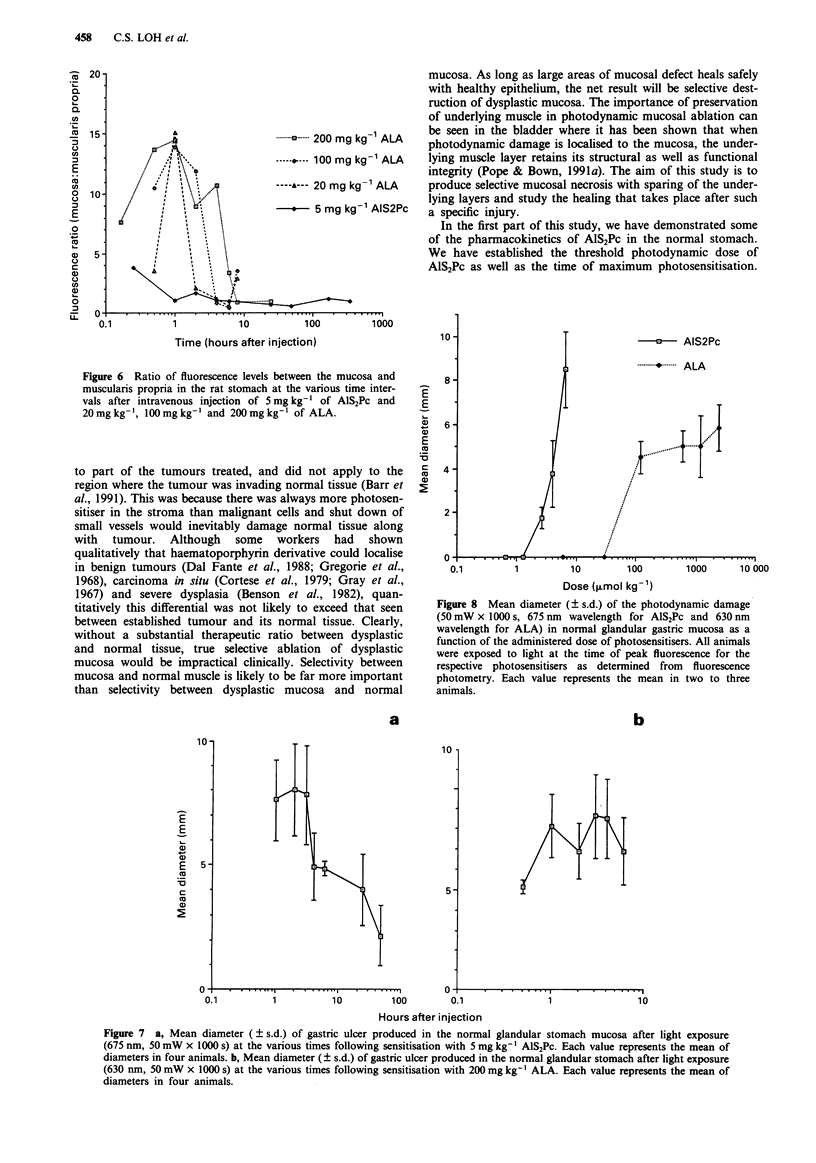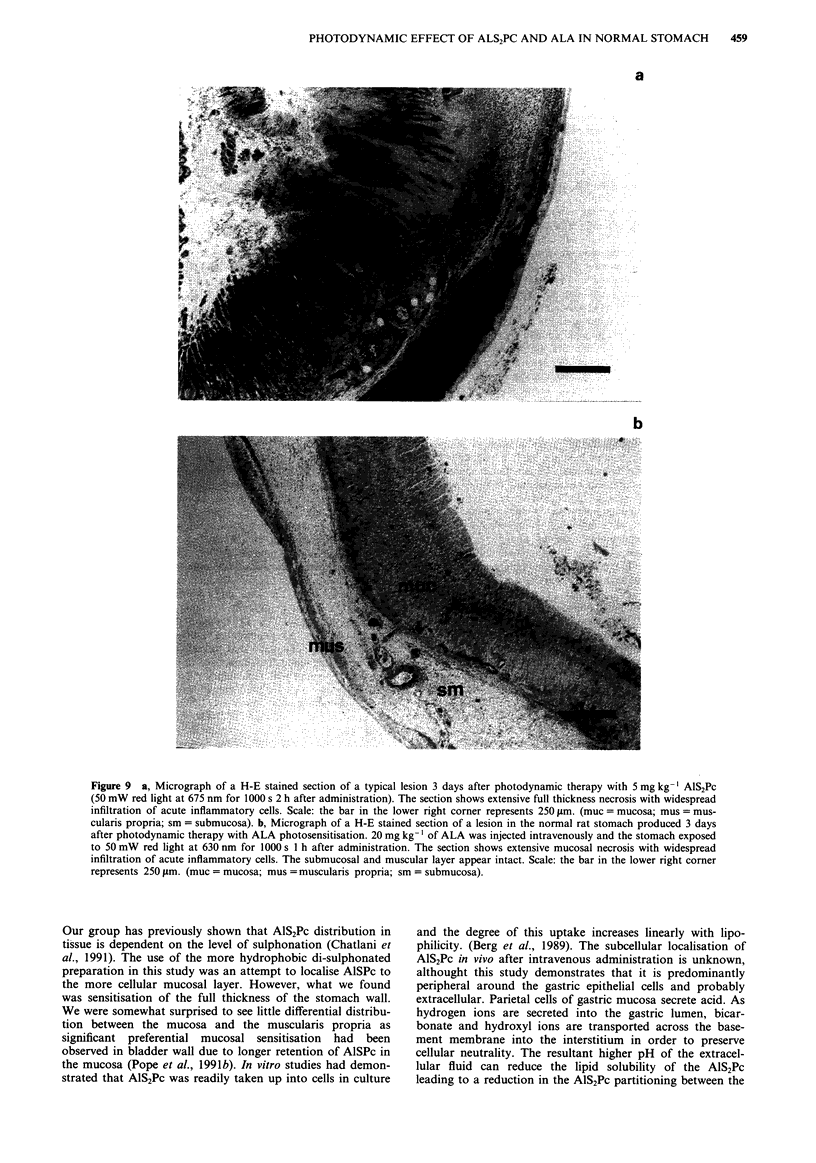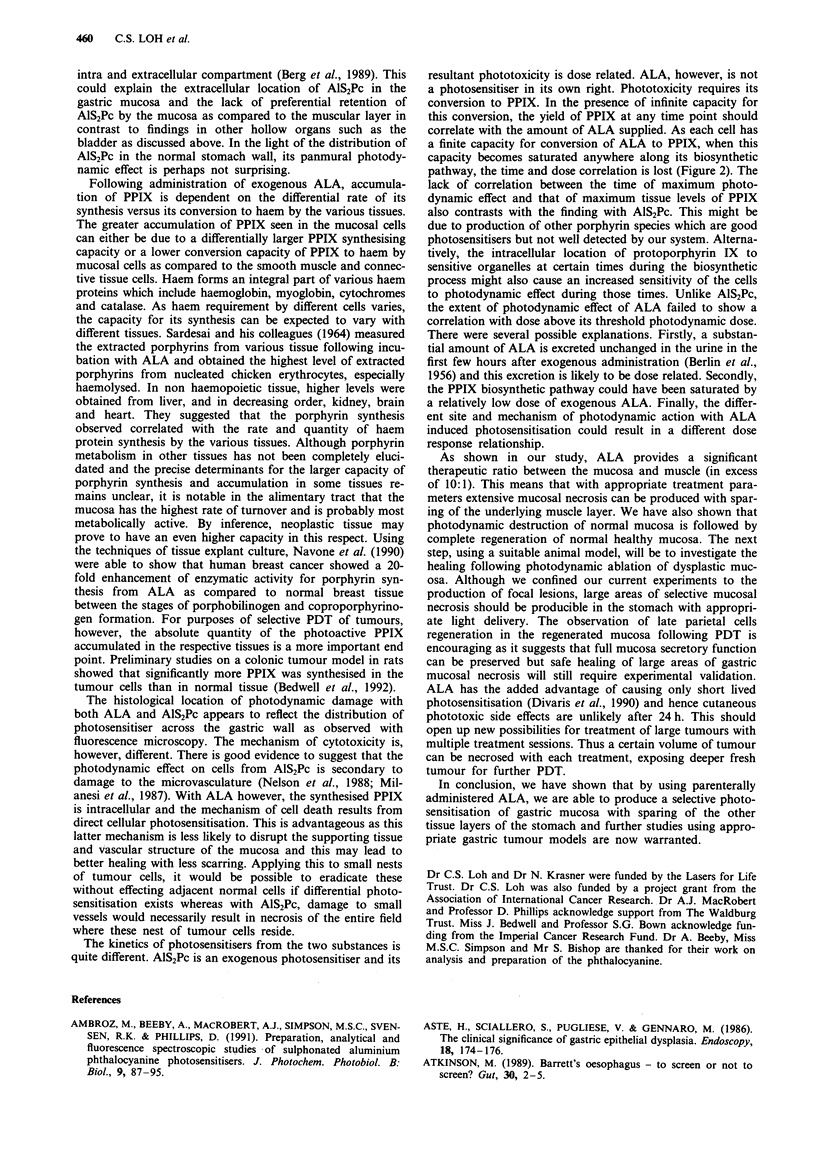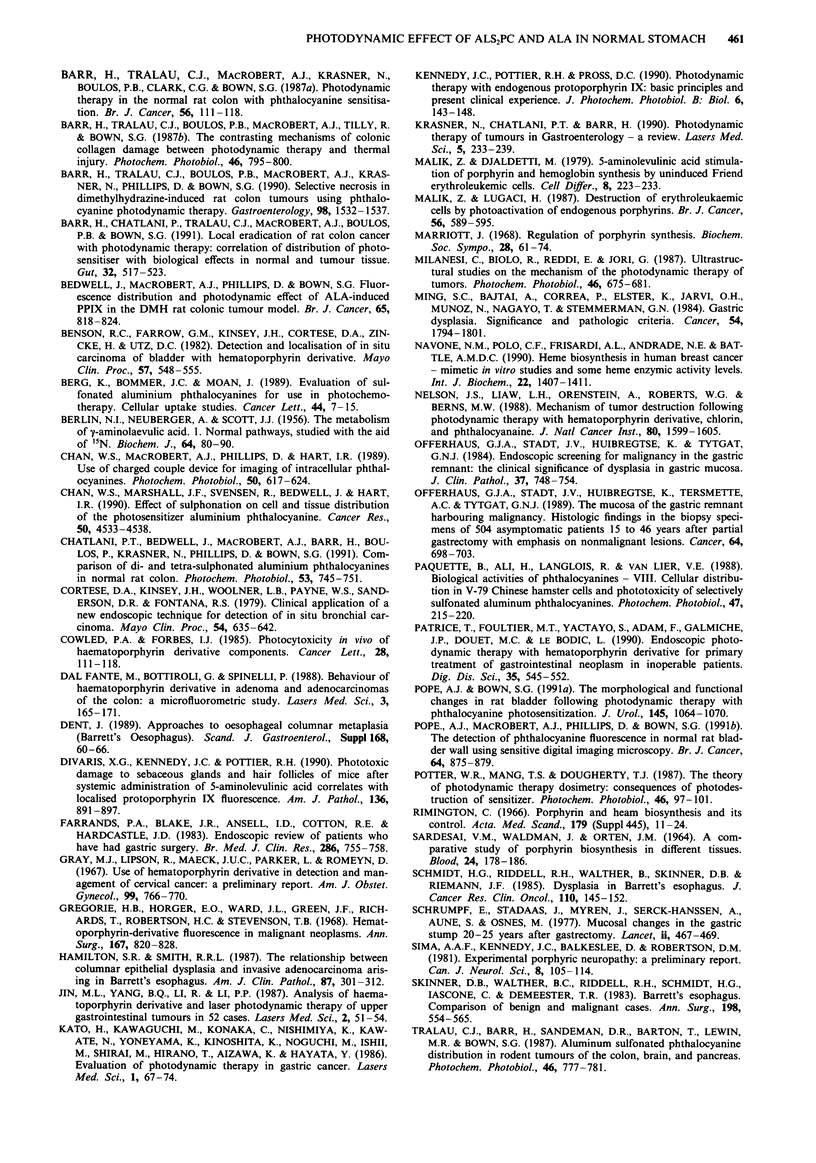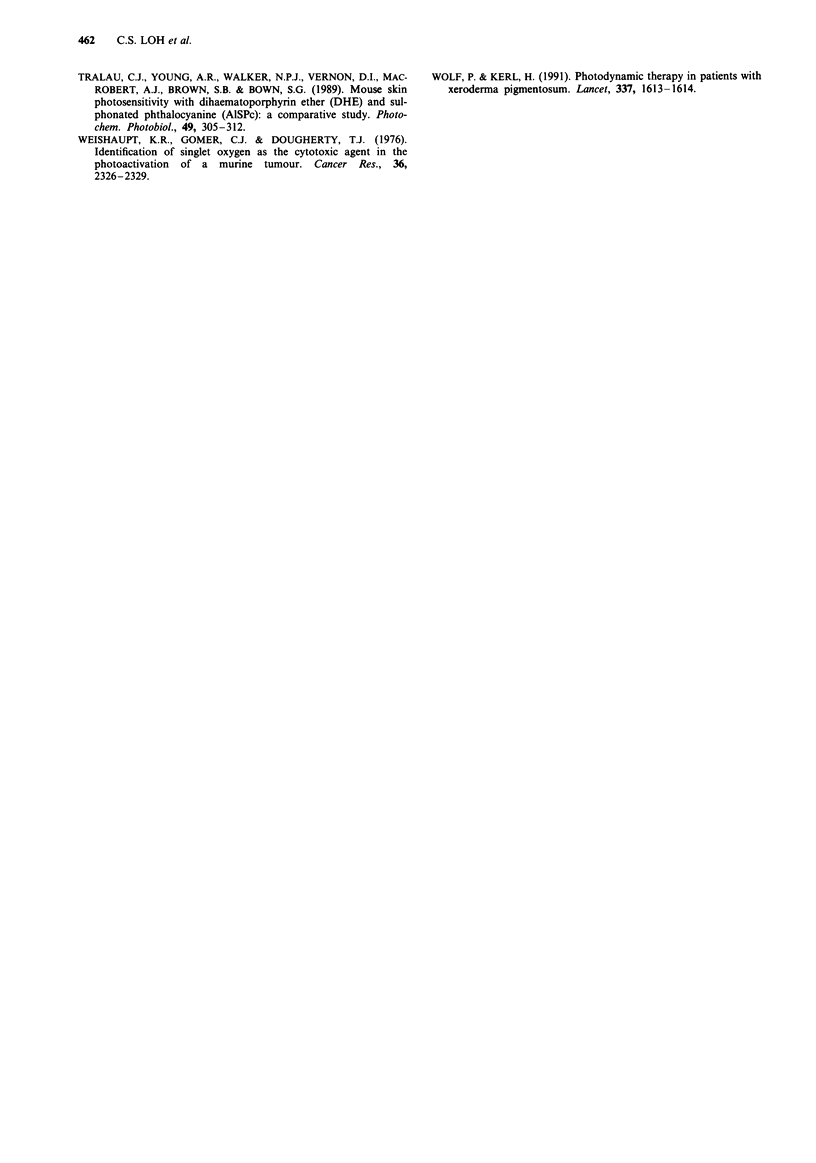# Photodynamic therapy of the normal rat stomach: a comparative study between di-sulphonated aluminium phthalocyanine and 5-aminolaevulinic acid.

**DOI:** 10.1038/bjc.1992.295

**Published:** 1992-09

**Authors:** C. S. Loh, J. Bedwell, A. J. MacRobert, N. Krasner, D. Phillips, S. G. Bown

**Affiliations:** Gastroenterology Unit, Walton Hospital, Liverpool, UK.

## Abstract

**Images:**


Br. J. Cancer (1992), 66, 452-462 © Macmillan Press Ltd., 1992

### Photodynamic therapy of the normal rat stomach: a comparative study between di-sulphonated aluminium phthalocyanine and 5-aminolaevulinic acid

C.S. Loh[1,2], J. Bedwell[2], A.J. MacRobert[3], N. Krasner[1], D. Phillips[3] & S.G. Bown[2]

[1]*Gastroenterology Unit, Walton Hospital, Rice Lane, Liverpool L9 1AE;* [2]*National Medical Laser Centre, Faculty of Clinical Sciences, University College London, The Rayne Institute, 5 University Street, London WC1E 6JJ;* [3]*Department of Chemistry, Imperial College of Science, Technology and Medicine, South Kensington, London SW7 2AY, UK.*

**Summary** Dysplasia in the upper gastrointestinal tract carries a risk of invasive malignant change. Surgical excision of the affected organ is the only treatment available. Photodynamic therapy has been shown to be promising in the treatment of early and superficial tumours and may be useful for the ablation of dysplastic mucosa. Because of the diffuse nature of the disease, such treatment would necessarily involve destruction of large areas of mucosa and it is desirable to confine its effect to the mucosa in order that safe healing can take place. By means of photometric fluorescence microscopy, we have studied the pattern of photosensitisation in the normal rat stomach using di-sulphonated aluminium phthalocyanine ($AlS_2Pc$) and 5-aminolaevulinic acid (ALA) as photosensitisers. $AlS_2Pc$ resulted in a panmural photosensitisation of the gastric wall with the highest level encountered in the submucosa. The mucosa and muscularis propria were sensitised to equal extent. Following light exposure, a full thickness damage resulted. ALA is a natural porphyrin precursor and exogenous administration gave rise to accumulation of protoporphyrin IX (PPIX) in the cells. The resultant pattern of photosensitisation was predominantly mucosal and its photodynamic effect was essentially confined to the mucosa. ALA produced a selective photosensitisation of the gastric mucosa for its photodynamic ablation with sparing the underlying tissue layers.

High grade epithelial dysplasia of gastric type mucosa whether in the stomach or oesophagus carries a risk of invasive malignant change (Farrands *et al.*, 1983; Skinner *et al.*, 1983; Offerhaus *et al.*, 1984; Schmidt *et al.*, 1985; Hamilton & Smith, 1987; Atkinson, 1989). In the stomach, dysplastic changes have been described in association with previous gastric surgery (Schrumpf *et al.*, 1977; Farrands *et al.*, 1983; Offerhaus *et al.*, 1984 & 1989), as well as gastric polyps (Aste *et al.*, 1986). In its severe form, the malignant potential is high (Ming *et al.*, 1984). In the oesophagus this condition often arises from Barrett's epithelium. There is no satisfactory treatment for this condition apart from surgical excision of the affected region of the organ concerned if this risk is significant (Skinner *et al.*, 1983; Ferrands *et al.*, 1983; Dent, 1989; Atkinson, 1989). Surgery is a major procedure and often not a viable option. Thermal laser ablation carries the risk of viscus perforation (Barr *et al.*, 1987*b*) and is not practical because of the diffuse and multicentric nature of dysplasia. Photodynamic therapy (PDT) of tumours involves the local activation of a preadministered photosensitiser by light of a specific wavelength matched to the absorption characteristic of the photosensitiser used. The activated photosensitiser subsequently give rise to production of cytotoxic singlet oxygen species (Weishaupt *et al.*, 1976). The mechanism of cell kill is by chemotoxicity and there is less likelihood of viscus perforation (Barr *et al.*, 1987*b*). PDT has been used to treat gastrointestinal tumours (Kato *et al.*, 1986; Jin *et al.*, 1987; Patrice *et al.*, 1990; Krasner *et al.*, 1990). Because of limited transmittance of light in tissue, it has been shown to be most effective in the treatment of small and early tumours (Kato *et al.*, 1986; Jin *et al.*, 1987; Krasner *et al.*, 1990). Epithelial dysplasia is an early superficial form of neoplastic lesion which is confined only to the mucosa and PDT may be a useful treatment modality for this condition. Due to its diffuse nature, treatment would necessarily involve destruction of large areas of mucosa. It is thus essential to limit the photodynamic effect to the mucosa to minimise complication and enhance prompt and safe healing. Photodynamic effect is local and only occurs when the products of the local light dose and photosensitiser concentration exceeds a threshold (Cowled & Forbes, 1985). In addition, because of photodegradation of photosensitiser by the activating light, the local tissue concentration of photosensitiser has to exceed a particular level irrespective of light dose before photodynamic action can take place (Potter *et al.*, 1987). When a sufficient differential photosensitiser distribution exist between mucosa and underlying structure, it is possible to produce a predominant photodynamic effect in the mucosa by keeping the concentration of photosensitiser in the mucosa above this threshold while that in the underlying tissue below it. This approach has been adopted experimentally in the bladder, and the resultant photodynamic effect causes minimal disruption of both the anatomical and functional integrity of the bladder while still achieving the desired objective of complete mucosal ablation (Pope & Bown, 1991*a*).

We have previously reported the efficacy of aluminium sulphonated phthalocyanine (AlSPc) as a photosensitiser in both normal tissue and tumours (Barr *et al.*, 1987*a*; Tralau *et al.*, 1987; Barr *et al.*, 1990) as well as its advantages over haematoporphyrin derivative with respect to cutaneous phototoxicity (Tralau *et al.*, 1989). We have further observed that PDT using AlSPc does not compromise the mechanical strength of the normal colon (Barr *et al.*, 1987*b*). Most of the PDT studies using AlSPc have been carried out using a mixture of compounds with different degree of sulphonation (range of 1 to 4 sulphonated groups with the average being 3.2). From recent studies, di-sulphonated phthalocyanine has been shown to be a more potent photosensitiser both *in vitro* and *in vivo* (Paquette *et al.*, 1988; Berg *et al.*, 1989; Chan *et al.*, 1990; Chatlani *et al.*, 1991). For these reasons, we have chosen to use the di-sulphonated aluminium phthalocyanine ($AlS_2Pc$) for this study.

ALA in itself is not a photosensitiser. It is a natural porphyrin precursor and its synthesis by living cells is the first committed step which will eventually lead to haem formation. In this biosynthetic pathway, the rate limiting step is that involved in the synthesis of ALA which is controlled by a regulatory feedback inhibition (Marriott, 1968; Rimington, 1966). By administering a large quantity of exogenous ALA

Correspondence: C.S. Loh.
Received 13 March 1992; and in revised form 14 May 1992.

both to *in vitro* systems as well as whole animals, it has been shown that the natural regulatory mechanism can become overloaded and as a result, porphyrin intermediates of the biosynthetic pathway, particularly protoporphyrin IX (PPIX), accumulate (Malik & Djaldetti, 1979; Sima *et al.*, 1981). PPIX is a potent photosensitiser. Malik & Lugaci (1987) and Kennedy's group (Divaris *et al.*, 1990) have shown that enough PPIX can be synthesised this way to produce a photodynamic effect both *in vitro* and *in vivo*. More recently, exogenous ALA applied topically has been shown to be effective in the photodynamic treatment of various cutaneous cancers (Kennedy *et al.*, 1990; Wolf & Kerl, 1991).

Although in theory all nucleated cells exhibiting aerobic metabolism capable of haem synthesis are liable to become photosensitised, Divaris found that following administration of exogenous ALA to mice, there was a marked difference in the level of photosensitisation in the various tissue structures in the skin as studied by fluorescence microscopy. The epidermal cells and cells of the pilosebaceous apparatus were markedly fluorescent as compared to the dermis (Divaris *et al.*, 1990). The same group has also reported that in the bladder and uterus, ALA administration resulted in preferential photosensitisation of the mucosa and endometrium over the other underlying structures of the respective organs. These findings prompted us to investigate ALA as a possible photosensitiser for photodynamic ablation of gastric mucosa.

### Materials and methods

### *Photosensitiser*

$AlS_2Pc$ was purified and analysed using high performance liquid chromatography (HPLC) at the Department of Chemistry, Imperial College of Science Technology and Medicine. The di-sulphonated fraction of $AlS_2Pc$ was separated from a mixture prepared by the oleum sulphonation of aluminium phthalocyanine chloride, using reverse phase liquid chromatography. This fraction contained a range of di-sulphonated isomers dominated by the most hydrophobic component which comprised $60 \pm 5\%$ of its integrated HPLC (Ambroz *et al.*, 1991). The photosensitiser was made up in 0.1 molar sodium hydroxide and phosphate buffered saline and administered intravenously via the tail vein. ALA was obtained as a hydrochloride (formular weight = 167.6) in 98% pure powder form from Sigma Chemical Company Limited (Poole, UK). It was dissolved in phosphate buffered saline for administration.

### *Animals*

All studies were performed on female Wistar rats supplied by the Imperial Cancer Research Fund. Their age ranged from 4 to 8 weeks and their weight ranged from 100 g to 200 g. Injections of photosensitisers were carried out under intramuscular Hypnorm (fentanyl and fluanisone) anaesthesia. The concentration of photosensitiser was adjusted to maintain the volume of injection between 0.3–0.5 ml to ensure accurate injection. Photodynamic therapy was carried out during laparotomy under intramuscular Hypnorm and diazepam anaesthesia.

### *Distributions of photosensitisers in the stomach*

This was studied by means of fluorescence microscopy and photometry. After administration of photosensitiser, animals were killed at a range of times from 15 min to 2 weeks. A small disc of stomach wall was excised from the glandular stomach along the greater curvature just distal to the limiting line and immediately frozen by submerging in a bath of isopentane (2-methylbutane) prechilled in liquid nitrogen. The snap frozen tissue samples were then stored in liquid nitrogen until sectioned. Tissue blocks were mounted on OCT medium (tissue tek II embedding compound, BDH) and 10 μm sections were cut using a Cryocut E microtome (Reichert-Jung). The slides were stored in a freezer at − 20°C and only allowed to thaw just prior to fluorescence microscopy. An inverted microscope (Olympus IMT-2) with epifluorescence and phase-contrast attachments was used as described previously (Chan *et al.*, 1989). Fluorescence excitation came from an 8 mW helium-neon laser (632.8 nm). The beam was delivered by a liquid light guide and through a 10 nm band-pass filter centred at 633 nm to remove extraneous light onto the dichroic mirror (Omega Optical Inc.) for epifluorescence study. The phthalocyanine fluorescence was detected between 665 and 700 nm using a combination of band-pass (Omega Optical Inc.) and long-pass (Schott RG665) filters. The fluorescence signal was detected by a highly sensitive cryogenically cooled CCD (charge-coupled device) camera (Wright Instruments, model 1, resolution 400 × 600 pixels) fitted to the microscope. This signal was processed by an IBM personal computer into a falsely colour-coded microscopic image of the section depicting the mean signal counts per pixel. The software also allowed quantitative analysis of the signal by calculating the mean fluorescence count and its standard deviation within any chosen area on the fluorescence image. Using a ten times objective of the microscope, a view of the entire cross section of the stomach was included. The mucosa, submucosa and muscularis propria were usually readily discernable on the fluorescence image. Three representative areas over each tissue layer at least 100 × 100 pixels in size were chosen for analysis on each section. Conventional light microscopy of the stained serial section of the specimen also helped to enable accurate identification of the various microscopic structures. As PPIX and $AlS_2Pc$ have very different fluorescence efficiency using 633 nm excitation, exposure time of the specimen to the exciting laser light was set to produce a comparable range of measurements (7.5 seconds for $AlS_2Pc$ and 25 seconds for ALA). Fluorescence was measured arbitrarily as counts per pixel (20 photoelectrons per count; quantum efficiency = 0.5 at this wavelength). The longer exposure time used for ALA resulted in higher tissue autofluorescence than that for $AlS_2Pc$. All fluorescence measurements were corrected for their respective autofluorescence (as measured on control specimens) of each respective layer of tissue with the respective exposure time for each photosensitiser. After fluorescence microscopy, specimens were fixed in formalin and stained with haemotoxylin and eosin. Both the falsely coloured coded fluorescence image and the light microscopic image of the subsequently stained section were photographed for comparison (Figure 3a, 3b, 4a and 4b). All studies with $AlS_2Pc$ were carried out using 5 mg $kg^{-1}$ (6.5 μmol $kg^{-1}$) of $AlS_2Pc$. As the conversion of ALA to the photoactive PPIX is dose dependent, a range of doses (20 mg $kg^{-1}$ (0.119 mmol $kg^{-1}$), 100 mg $kg^{-1}$ (0.597 mmol $kg^{-1}$) and 200 mg $kg^{-1}$ (1.193 mmol $kg^{-1}$) were employed.

### *Photodynamic therapy*

The light source used was a pulsed (12 kHz) copper vapour pumped dye laser (Oxford Lasers). In the $AlS_2Pc$ group, the output was tuned to 675 nm (peak absorption for $AlS_2Pc$) and delivered via a 200 μm fibre threaded into the stomach through the forestomach and held just touching the mucosa of the glandular stomach. The fibre was maintained at approximately 90° to the mucosal surface. The rest of the abdominal viscera were shielded from forward light scatter by interposition of a piece of opaque paper. Only one point was treated in each animal. Power output from the fibre tip was 50 mW and the total irradiation time 1000 sec giving a total energy delivery of 50 J per animal. In one sub-group, all animals were sensitised with 5 mg $kg^{-1}$ of $AlS_2Pc$ and then exposed to laser light at a range of times from 1 to 48 h following sensitisation. In the other sub-group, animals were sensitised with $AlS_2Pc$ at a range of doses from 0.5 mg $kg^{-1}$ to 5 mg $kg^{-1}$ and then exposed to laser light 2 h later. In the ALA group, the laser was tuned to 630 nm and the same power and exposure time were used. Two sub-groups of animals were treated with ALA photosensitisation. In one sub-group, all animals were given 200 mg $kg^{-1}$ of ALA and light exposure was effected at a range of time from 30 min to 8 h. In the other sub-group, animals were sensitised with different doses of ALA (1 mg $kg^{-1}$, 5 mg $kg^{-1}$, 20 mg $kg^{-1}$, 100 mg $kg^{-1}$, 200 mg $kg^{-1}$ and 400 mg $kg^{-1}$) and then exposed to laser light at the time of peak photosensitisation of the respective doses as determined from fluorescence photometry. Fluorescence photometry was not carried out with 1 mg $kg^{-1}$ and 5 mg $kg^{-1}$ of ALA because with these doses, the fluorescence yield was too low relative to the background tissue fluorescence to provide sufficient contrast for detection using our system. Control unsensitised animals were irradiated using similar parameters to exclude thermal effects. Treated areas were marked with two silk sutures placed along the greater curve at 1 cm proximal and 1 cm distal to the point of contact of the laser fibre to ease subsequent identification. Animals were allowed to recover and kept in standard laboratory conditions until sacrificed at 72 h. On killing the animal, the stomach was immediately excised and opened along the lesser curve for macroscopic inspection. The specimens were laid out on a piece of card and the size of the PDT induced lesions were determined by taking the mean of the longest diameter and the broadest diameter of the lesion (Barr *et al*., 1987*a*). The specimen was then fixed in formalin and prepared for conventional light microscopy.

### Results

### *Fluorescence photometry*

Fluorescence spectroscopy using a Perkin-Elmer LS-5B spectrofluorimeter (excitation at 400 nm) of an *ex vivo* specimen of stomach from a rat sensitised with 200 mg $kg^{-1}$ of ALA was carried out and the spectrum obtained was found to be consistent with the fluorescence emission spectrum of PPIX as also found by Divaris *et al*. (1990). Following administration of 5 mg $kg^{-1}$ of $AlS_2Pc$, fluorescence reached a peak at 1 h and rapidly declined in the first 48 h (Figure 1a). By 2 weeks, the fluorescence signal approached that of the control specimen. At all time points, the highest uptake of $AlS_2Pc$ was seen in the submucosa. Mean uptake by the submucosa was approximately twice that of the mucosa and muscularis propria. With 200 mg $kg^{-1}$ of ALA, the fluorescence signal in the mucosal layer rose rapidly to a peak at 3 h while signal over the other layers rose much less (Figure 1b). Peak fluorescence was achieved earlier with the 20 mg $kg^{-1}$ of ALA as compared to higher doses and the trend suggested an earlier fluorescence peak with 100 mg $kg^{-1}$ as compared to 200 mg $kg^{-1}$ (Figure 2). Although the level of maximum fluorescence increased with the dose of ALA administered, this relationship was not a linear one and the peak fluorescence level achieved with 200 mg $kg^{-1}$ of ALA was only marginally higher than that with 100 mg $kg^{-1}$. Fluorescence declined very rapidly and almost reaching background level by 6 to 8 h.

The microscopic distribution of fluorescence after administration of 5 mg $kg^{-1}$ of $AlS_2Pc$ is represented in Figure 3a and b. Highest levels of fluorescence were seen in the submucosal layer and particularly around blood vessels. Fluorescence levels in the mucosa and muscularis were comparable and both lower than that found in the submucosa. With ALA however, the resultant PPIX fluorescence was predominantly over the mucosa (Figure 4a and b) with very little fluorescence seen over the submucosa and muscularis propria. At higher magnification, $AlS_2Pc$ fluorescence was highest around the periphery of the epithelial cells in the mucosa suggesting that $AlS_2Pc$ was largely extracellular (Figure 5a).

**Figure 2** Mean level of fluorescence (± s.d.) of the gastric mucosa after intravenous administration of 20 mg $kg^{-1}$, 100 mg $kg^{-1}$ and 200 mg $kg^{-1}$ of ALA as a function of time. All values have been corrected for tissue autofluorescence. Value at each time point represents the mean in three or four animals.

**Figure 1** Mean level of fluorescence (± s.d.) of the layers of the gastric wall after intravenous administration of 5 mg $kg^{-1}$ of $AlS_2Pc$ **a**, or 200 mg $kg^{-1}$ ALA **b**, as a function of time. All values have been corrected for tissue autofluorescence. Value at each time point represents the mean in three or four animals.

**Figure 3** **a**, Fluorescence image of a frozen section of gastric wall 4 h after intravenous administration of 5 mg $kg^{-1}$ of $AlS_2Pc$. The upper colour bar represents the fluorescence scale (Black = 25 counts per pixel; White = 256 counts per pixel). Scale: the bar in the right bottom corner represents 100 $\mu$m. (muc = mucosa; mus = muscularis propria; sm = submucosa). **b**, Micrograph of section in **a** after H-E staining showing the corresponding mucosal, submucosal and muscular layer. Scale: the bar in the right bottom corner represents 100 $\mu$m. (muc = mucosa; mus = muscularis propria; sm = submucosa).

ALA induced PPIX fluorescence was, however, intracellular in location and mainly perinuclear (Figure 5b).

The relative level of fluorescence of the mucosa and muscularis propria achieved following administration of both compounds differed markedly. With $AlS_2Pc$, apart from immediately following administration when a significant quantity of photosensitiser was still in the intravascular compartment, the ratio of fluorescence between the mucosa and the muscularis propria remained at approximately 1 throughout. In contrast, after administration of ALA, the ratio of fluorescence level between mucosa and muscle varied with time (Figure 6). With all doses of ALA, this ratio rose to a peak in excess of 14 one hour after administration. With 20 mg $kg^{-1}$, of ALA, this ratio fell back rapidly to almost unity at 2 h. When higher doses of ALA were given, the high fluorescence ratio was sustained over a longer duration and with the dose of 200 mg $kg^{-1}$, did not reach unity until 8 h after administration.

*Photodynamic therapy*

No macroscopic lesion was seen in the control groups although on close microscopic scrutiny, a small area of mucosal necrosis comparable to the diameter of the fibre could be found. In contrast, photodynamic lesions were macroscopically obvious if present. After photosensitisation with 5 mg $kg^{-1}$ of $AlS_2Pc$, maximum damage occurred when animals were exposed to light between 1 and 3 h after sensitisation (Figure 7a) which correlated relatively well with the time peak of $AlS_2Pc$ fluorescence. Using 200 mg $kg^{-1}$ of ALA, apart from a lesser extent of damage produced when light exposure occurred at ½ hour after administration, the extent

**Figure 4** **a**, Fluorescence image of a frozen section of gastric wall 3 h after intravenous administration of 200 mg $kg^{-1}$ of ALA. The upper colour bar represents the fluorescence scale (Black = 25 counts per pixel; White = 256 counts per pixel). The mucosal layer is brightly fluorescent while fluorescence levels in all the other layers of the gastric wall are little above background. Scale: the bar in the right bottom corner represents 100 μm. (muc = mucosa; mus = muscularis propria; sm = submucosa). **b**, Micrograph of section in **a** after H-E staining showing the corresponding mucosal, submucosal and muscular layer. Scale: the bar in the right bottom corner represents 100 μm. (muc = mucosa; mus = muscularis propria; sm = submucosa).

of damage appeared to plateau off subsequently when light exposure occurred between 1 and 6 h after administration (Figure 7b).

The extent of photodynamic damage produced with $AlS_2Pc$ photosensitisation varied with the dose administered. No photodynamic lesion was produced at the sensitiser dose of 0.5 or 1 mg $kg^{-1}$. Above 1 mg $kg^{-1}$, the size of the lesion produced appeared to correlate with increasing dose of $AlS_2Pc$. This dose effect was not seen with ALA photosensitisation. No PDT damage was evident when the animals were sensitised with 1 mg $kg^{-1}$ or 5 mg $kg^{-1}$ of ALA. However, the mean diameter of the PDT lesion remained relatively constant despite a 20 times increase in the dose of ALA given from 20 mg $kg^{-1}$ to 400 mg $kg^{-1}$ (Figure 8). The threshold dose of ALA for photodynamic effect in the stomach lies between 5 mg $kg^{-1}$ and 20 mg $kg^{-1}$.

Histology at 72 h after PDT with 5 mg $kg^{-1}$ of $AlS_2Pc$ showed a full thickness necrosis of the gastric wall with widespread infiltration of acute inflammatory cells (Figure 9a). The same transmural necrosis was seen with doses of 3 mg $kg^{-1}$ and 2 mg $kg^{-1}$. Two weeks after PDT, the dead tissue has been demolished either by sloughing or resorption leaving a full thickness defect which was bridged by extensive deposition of scar tissue and new collagen on the serosal aspect. The mucosal defect was re-epithelialised initially with mucus secreting glandular epithelium but by 16 weeks after PDT, there were parietal cells and smooth muscle regeneration. No microscopic evidence of photodynamic damage was

**Figure 5** **a**, Grey scale fluorescence micrograph of a frozen section of gastric mucosa 4 h after intravenous administration of 5 mg $kg^{-1}$ of $AlS_2Pc$ showing high level of fluorescence mainly around the periphery of the epithelial cells. Scale: the bar in the lower right corner represents 25 $\mu m$. **b**, Grey scale fluorescence micrograph of a frozen section of gastric mucosa 1 h after intravenous administration of 200 mg $kg^{-1}$ ALA showing high level of fluorescence mainly inside the cells but outside the nuclei. Scale: the bar in the lower right corner represents 25 $\mu m$.

seen with 1 mg $kg^{-1}$ of $AlS_2Pc$. The photodynamic effect of ALA photosensitisation was predominantly confined to the mucosa with extensive necrosis of mucosal epithelial cells. There was some damage to the muscularis propria when a dose of 200 mg $kg^{-1}$ was used but the layer remain viable. With the dose of 20 mg $kg^{-1}$ however, the submucosa and muscularis were hardly affected (Figure 9b) and the resultant healing involve minimal scar tissue formation.

### Discussion

Dysplasia in the alimentary tract is a difficult clinical problem. Although its malignant potential is well known, many clinicians are reluctant to advise excisional surgery in the absence of invasive malignant change. PDT may be an effective but less invasive treatment for this condition. The ideal goal would be the selective destruction of only the dysplastic mucosa. Barr *et al.* (1990) had shown that with AlSPc truly selective tumour necrosis (i.e. necrosis of tumour tissue but not the adjacent normal tissue from which the tumour arise) could be produced by judicious manipulation of treatment parameters in such a way that the photosensitiser concentration in the normal tissue fell below the photodynamic threshold while that in the tumour tissue remained above it. The volume of tumour necrosis produced was however very small. This was due to the small therapeutic ratio of conventional photosensitiser (2:1) between tumour and adjacent normal tissue (Tralau *et al.*, 1987). In addition, this true selectivity could only be applied

**Figure 6** Ratio of fluorescence levels between the mucosa and muscularis propria in the rat stomach at the various time intervals after intravenous injection of $5\,mg\,kg^{-1}$ of $AlS_2Pc$ and $20\,mg\,kg^{-1}$, $100\,mg\,kg^{-1}$ and $200\,mg\,kg^{-1}$ of ALA.

to part of the tumours treated, and did not apply to the region where the tumour was invading normal tissue (Barr *et al.*, 1991). This was because there was always more photosensitiser in the stroma than malignant cells and shut down of small vessels would inevitably damage normal tissue along with tumour. Although some workers had shown qualitatively that haematoporphyrin derivative could localise in benign tumours (Dal Fante *et al.*, 1988; Gregorie *et al.*, 1968), carcinoma *in situ* (Cortese *et al.*, 1979; Gray *et al.*, 1967) and severe dysplasia (Benson *et al.*, 1982), quantitatively this differential was not likely to exceed that seen between established tumour and its normal tissue. Clearly, without a substantial therapeutic ratio between dysplastic and normal tissue, true selective ablation of dysplastic mucosa would be impractical clinically. Selectivity between mucosa and normal muscle is likely to be far more important than selectivity between dysplastic mucosa and normal mucosa. As long as large areas of mucosal defect heals safely with healthy epithelium, the net result will be selective destruction of dysplastic mucosa. The importance of preservation of underlying muscle in photodynamic mucosal ablation can be seen in the bladder where it has been shown that when photodynamic damage is localised to the mucosa, the underlying muscle layer retains its structural as well as functional integrity (Pope & Bown, 1991*a*). The aim of this study is to produce selective mucosal necrosis with sparing of the underlying layers and study the healing that takes place after such a specific injury.

In the first part of this study, we have demonstrated some of the pharmacokinetics of $AlS_2Pc$ in the normal stomach. We have established the threshold photodynamic dose of $AlS_2Pc$ as well as the time of maximum photosensitisation.

**Figure 8** Mean diameter (± s.d.) of the photodynamic damage ($50\,mW \times 1000\,s$, 675 nm wavelength for $AlS_2Pc$ and 630 nm wavelength for ALA) in normal glandular gastric mucosa as a function of the administered dose of photosensitisers. All animals were exposed to light at the time of peak fluorescence for the respective photosensitisers as determined from fluorescence photometry. Each value represents the mean in two to three animals.

**Figure 7** **a**, Mean diameter (± s.d.) of gastric ulcer produced in the normal glandular stomach mucosa after light exposure (675 nm, $50\,mW \times 1000\,s$) at the various times following sensitisation with $5\,mg\,kg^{-1}$ $AlS_2Pc$. Each value represents the mean of diameters in four animals. **b**, Mean diameter (± s.d.) of gastric ulcer produced in the normal glandular stomach after light exposure (630 nm, $50\,mW \times 1000\,s$) at the various times following sensitisation with $200\,mg\,kg^{-1}$ ALA. Each value represents the mean of diameters in four animals.

**Figure 9** **a**, Micrograph of a H-E stained section of a typical lesion 3 days after photodynamic therapy with 5 mg $kg^{-1}$ $AlS_2Pc$ (50 mW red light at 675 nm for 1000 s 2 h after administration). The section shows extensive full thickness necrosis with widespread infiltration of acute inflammatory cells. Scale: the bar in the lower right corner represents 250 μm. (muc = mucosa; mus = muscularis propria; sm = submucosa). **b**, Micrograph of a H-E stained section of a lesion in the normal rat stomach produced 3 days after photodynamic therapy with ALA photosensitisation. 20 mg $kg^{-1}$ of ALA was injected intravenously and the stomach exposed to 50 mW red light at 630 nm for 1000 s 1 h after administration. The section shows extensive mucosal necrosis with widespread infiltration of acute inflammatory cells. The submucosal and muscular layer appear intact. Scale: the bar in the lower right corner represents 250 μm. (muc = mucosa; mus = muscularis propria; sm = submucosa).

Our group has previously shown that $AlS_2Pc$ distribution in tissue is dependent on the level of sulphonation (Chatlani *et al.*, 1991). The use of the more hydrophobic di-sulphonated preparation in this study was an attempt to localise AlSPc to the more cellular mucosal layer. However, what we found was sensitisation of the full thickness of the stomach wall. We were somewhat surprised to see little differential distribution between the mucosa and the muscularis propria as significant preferential mucosal sensitisation had been observed in bladder wall due to longer retention of AlSPc in the mucosa (Pope *et al.*, 1991*b*). *In vitro* studies had demonstrated that $AlS_2Pc$ was readily taken up into cells in culture and the degree of this uptake increases linearly with lipophilicity. (Berg *et al.*, 1989). The subcellular localisation of $AlS_2Pc$ *in vivo* after intravenous administration is unknown, althought this study demonstrates that it is predominantly peripheral around the gastric epithelial cells and probably extracellular. Parietal cells of gastric mucosa secrete acid. As hydrogen ions are secreted into the gastric lumen, bicarbonate and hydroxyl ions are transported across the basement membrane into the interstitium in order to preserve cellular neutrality. The resultant higher pH of the extracellular fluid can reduce the lipid solubility of the $AlS_2Pc$ leading to a reduction in the $AlS_2Pc$ partitioning between the intra and extracellular compartment (Berg *et al.*, 1989). This could explain the extracellular location of $AlS_2Pc$ in the gastric mucosa and the lack of preferential retention of $AlS_2Pc$ by the mucosa as compared to the muscular layer in contrast to findings in other hollow organs such as the bladder as discussed above. In the light of the distribution of $AlS_2Pc$ in the normal stomach wall, its panmural photodynamic effect is perhaps not surprising.

Following administration of exogenous ALA, accumulation of PPIX is dependent on the differential rate of its synthesis versus its conversion to haem by the various tissues. The greater accumulation of PPIX seen in the mucosal cells can either be due to a differentially larger PPIX synthesising capacity or a lower conversion capacity of PPIX to haem by mucosal cells as compared to the smooth muscle and connective tissue cells. Haem forms an integral part of various haem proteins which include haemoglobin, myoglobin, cytochromes and catalase. As haem requirement by different cells varies, the capacity for its synthesis can be expected to vary with different tissues. Sardesai and his colleagues (1964) measured the extracted porphyrins from various tissue following incubation with ALA and obtained the highest level of extracted porphyrins from nucleated chicken erythrocytes, especially haemolysed. In non haemopoietic tissue, higher levels were obtained from liver, and in decreasing order, kidney, brain and heart. They suggested that the porphyrin synthesis observed correlated with the rate and quantity of haem protein synthesis by the various tissues. Although porphyrin metabolism in other tissues has not been completely elucidated and the precise determinants for the larger capacity of porphyrin synthesis and accumulation in some tissues remains unclear, it is notable in the alimentary tract that the mucosa has the highest rate of turnover and is probably most metabolically active. By inference, neoplastic tissue may prove to have an even higher capacity in this respect. Using the techniques of tissue explant culture, Navone *et al.* (1990) were able to show that human breast cancer showed a 20-fold enhancement of enzymatic activity for porphyrin synthesis from ALA as compared to normal breast tissue between the stages of porphobilinogen and coproporphyrinogen formation. For purposes of selective PDT of tumours, however, the absolute quantity of the photoactive PPIX accumulated in the respective tissues is a more important end point. Preliminary studies on a colonic tumour model in rats showed that significantly more PPIX was synthesised in the tumour cells than in normal tissue (Bedwell *et al.*, 1992).

The histological location of photodynamic damage with both ALA and $AlS_2Pc$ appears to reflect the distribution of photosensitiser across the gastric wall as observed with fluorescence microscopy. The mechanism of cytotoxicity is, however, different. There is good evidence to suggest that the photodynamic effect on cells from $AlS_2Pc$ is secondary to damage to the microvasculature (Nelson *et al.*, 1988; Milanesi *et al.*, 1987). With ALA however, the synthesised PPIX is intracellular and the mechanism of cell death results from direct cellular photosensitisation. This is advantageous as this latter mechanism is less likely to disrupt the supporting tissue and vascular structure of the mucosa and this may lead to better healing with less scarring. Applying this to small nests of tumour cells, it would be possible to eradicate these without effecting adjacent normal cells if differential photosensitisation exists whereas with $AlS_2Pc$, damage to small vessels would necessarily result in necrosis of the entire field where these nest of tumour cells reside.

The kinetics of photosensitisers from the two substances is quite different. $AlS_2Pc$ is an exogenous photosensitiser and its resultant phototoxicity is dose related. ALA, however, is not a photosensitiser in its own right. Phototoxicity requires its conversion to PPIX. In the presence of infinite capacity for this conversion, the yield of PPIX at any time point should correlate with the amount of ALA supplied. As each cell has a finite capacity for conversion of ALA to PPIX, when this capacity becomes saturated anywhere along its biosynthetic pathway, the time and dose correlation is lost (Figure 2). The lack of correlation between the time of maximum photodynamic effect and that of maximum tissue levels of PPIX also contrasts with the finding with $AlS_2Pc$. This might be due to production of other porphyrin species which are good photosensitisers but not well detected by our system. Alternatively, the intracellular location of protoporphyrin IX to sensitive organelles at certain times during the biosynthetic process might also cause an increased sensitivity of the cells to photodynamic effect during those times. Unlike $AlS_2Pc$, the extent of photodynamic effect of ALA failed to show a correlation with dose above its threshold photodynamic dose. There were several possible explanations. Firstly, a substantial amount of ALA is excreted unchanged in the urine in the first few hours after exogenous administration (Berlin *et al.*, 1956) and this excretion is likely to be dose related. Secondly, the PPIX biosynthetic pathway could have been saturated by a relatively low dose of exogenous ALA. Finally, the different site and mechanism of photodynamic action with ALA induced photosensitisation could result in a different dose response relationship.

As shown in our study, ALA provides a significant therapeutic ratio between the mucosa and muscle (in excess of 10:1). This means that with appropriate treatment parameters extensive mucosal necrosis can be produced with sparing of the underlying muscle layer. We have also shown that photodynamic destruction of normal mucosa is followed by complete regeneration of normal healthy mucosa. The next step, using a suitable animal model, will be to investigate the healing following photodynamic ablation of dysplastic mucosa. Although we confined our current experiments to the production of focal lesions, large areas of selective mucosal necrosis should be producible in the stomach with appropriate light delivery. The observation of late parietal cells regeneration in the regenerated mucosa following PDT is encouraging as it suggests that full mucosa secretory function can be preserved but safe healing of large areas of gastric mucosal necrosis will still require experimental validation. ALA has the added advantage of causing only short lived photosensitisation (Divaris *et al.*, 1990) and hence cutaneous phototoxic side effects are unlikely after 24 h. This should open up new possibilities for treatment of large tumours with multiple treatment sessions. Thus a certain volume of tumour can be necrosed with each treatment, exposing deeper fresh tumour for further PDT.

In conclusion, we have shown that by using parenterally administered ALA, we are able to produce a selective photosensitisation of gastric mucosa with sparing of the other tissue layers of the stomach and further studies using appropriate gastric tumour models are now warranted.

Dr C.S. Loh and Dr N. Krasner were funded by the Lasers for Life Trust. Dr C.S. Loh was also funded by a project grant from the Association of International Cancer Research. Dr A.J. MacRobert and Professor D. Phillips acknowledge support from The Waldburg Trust. Miss J. Bedwell and Professor S.G. Bown acknowledge funding from the Imperial Cancer Research Fund. Dr A. Beeby, Miss M.S.C. Simpson and Mr S. Bishop are thanked for their work on analysis and preparation of the phthalocyanine.

### References

AMBROZ, M., BEEBY, A., MACROBERT, A.J., SIMPSON, M.S.C., SVENSEN, R.K. & PHILLIPS, D. (1991). Preparation, analytical and fluorescence spectroscopic studies of sulphonated aluminium phthalocyanine photosensitisers. *J. Photochem. Photobiol. B: Biol.*, **9**, 87–95.

ASTE, H., SCIALLERO, S., PUGLIESE, V. & GENNARO, M. (1986). The clinical significance of gastric epithelial dysplasia. *Endoscopy*, **18**, 174–176.

ATKINSON, M. (1989). Barrett's oesophagus – to screen or not to screen? *Gut*, **30**, 2–5.

BARR, H., TRALAU, C.J., MACROBERT, A.J., KRASNER, N., BOULOS, P.B., CLARK, C.G. & BOWN, S.G. (1987*a*). Photodynamic therapy in the normal rat colon with phthalocyanine sensitisation. *Br. J. Cancer*, **56**, 111–118.

BARR, H., TRALAU, C.J., BOULOS, P.B., MACROBERT, A.J., TILLY, R. & BOWN, S.G. (1987*b*). The contrasting mechanisms of colonic collagen damage between photodynamic therapy and thermal injury. *Photochem. Photobiol.*, **46**, 795–800.

BARR, H., TRALAU, C.J., BOULOS, P.B., MACROBERT, A.J., KRASNER, N., PHILLIPS, D. & BOWN, S.G. (1990). Selective necrosis in dimethylhydrazine-induced rat colon tumours using phthalocyanine photodynamic therapy. *Gastroenterology*, **98**, 1532–1537.

BARR, H., CHATLANI, P., TRALAU, C.J., MACROBERT, A.J., BOULOS, P.B. & BOWN, S.G. (1991). Local eradication of rat colon cancer with photodynamic therapy: correlation of distribution of photosensitiser with biological effects in normal and tumour tissue. *Gut*, **32**, 517–523.

BEDWELL, J., MACROBERT, A.J., PHILLIPS, D. & BOWN, S.G. Fluorescence distribution and photodynamic effect of ALA-induced PPIX in the DMH rat colonic tumour model. *Br. J. Cancer*, **65**, 818–824.

BENSON, R.C., FARROW, G.M., KINSEY, J.H., CORTESE, D.A., ZINCKE, H. & UTZ, D.C. (1982). Detection and localisation of in situ carcinoma of bladder with hematoporphyrin derivative. *Mayo Clin. Proc.*, **57**, 548–555.

BERG, K., BOMMER, J.C. & MOAN, J. (1989). Evaluation of sulfonated aluminium phthalocyanines for use in photochemotherapy. Cellular uptake studies. *Cancer Lett.*, **44**, 7–15.

BERLIN, N.I., NEUBERGER, A. & SCOTT, J.J. (1956). The metabolism of γ-aminolaevulic acid. 1. Normal pathways, studied with the aid of $^{15}N$. *Biochem. J.*, **64**, 80–90.

CHAN, W.S., MACROBERT, A.J., PHILLIPS, D. & HART, I.R. (1989). Use of charged couple device for imaging of intracellular phthalocyanines. *Photochem. Photobiol.*, **50**, 617–624.

CHAN, W.S., MARSHALL, J.F., SVENSEN, R., BEDWELL, J. & HART, I.R. (1990). Effect of sulphonation on cell and tissue distribution of the photosensitizer aluminium phthalocyanine. *Cancer Res.*, **50**, 4533–4538.

CHATLANI, P.T., BEDWELL, J., MACROBERT, A.J., BARR, H., BOULOS, P., KRASNER, N., PHILLIPS, D. & BOWN, S.G. (1991). Comparison of di- and tetra-sulphonated aluminium phthalocyanines in normal rat colon. *Photochem. Photobiol.*, **53**, 745–751.

CORTESE, D.A., KINSEY, J.H., WOOLNER, L.B., PAYNE, W.S., SANDERSON, D.R. & FONTANA, R.S. (1979). Clinical application of a new endoscopic technique for detection of in situ bronchial carcinoma. *Mayo Clin. Proc.*, **54**, 635–642.

COWLED, P.A. & FORBES, I.J. (1985). Photocytoxicity *in vivo* of haematoporphyrin derivative components. *Cancer Lett.*, **28**, 111–118.

DAL FANTE, M., BOTTIROLI, G. & SPINELLI, P. (1988). Behaviour of haematoporphyrin derivative in adenoma and adenocarcinomas of the colon: a microfluorometric study. *Lasers Med. Sci.*, **3**, 165–171.

DENT, J. (1989). Approaches to oesophageal columnar metaplasia (Barrett's Oesophagus). *Scand. J. Gastroenterol.*, **Suppl 168**, 60–66.

DIVARIS, X.G., KENNEDY, J.C. & POTTIER, R.H. (1990). Phototoxic damage to sebaceous glands and hair follicles of mice after systemic administration of 5-aminolevulinic acid correlates with localised protoporphyrin IX fluorescence. *Am. J. Pathol.*, **136**, 891–897.

FARRANDS, P.A., BLAKE, J.R., ANSELL, I.D., COTTON, R.E. & HARDCASTLE, J.D. (1983). Endoscopic review of patients who have had gastric surgery. *Br. Med. J. Clin. Res.*, **286**, 755–758.

GRAY, M.J., LIPSON, R., MAECK, J.U.C., PARKER, L. & ROMEYN, D. (1967). Use of hematoporphyrin derivative in detection and management of cervical cancer: a preliminary report. *Am. J. Obstet. Gynecol.*, **99**, 766–770.

GREGORIE, H.B., HORGER, E.O., WARD, J.L., GREEN, J.F., RICHARDS, T., ROBERTSON, H.C. & STEVENSON, T.B. (1968). Hematoporphyrin-derivative fluorescence in malignant neoplasms. *Ann. Surg.*, **167**, 820–828.

HAMILTON, S.R. & SMITH, R.R.L. (1987). The relationship between columnar epithelial dysplasia and invasive adenocarcinoma arising in Barrett's esophagus. *Am. J. Clin. Pathol.*, **87**, 301–312.

JIN, M.L., YANG, B.Q., LI, R. & LI, P.P. (1987). Analysis of haematoporphyrin derivative and laser photodynamic therapy of upper gastrointestinal tumours in 52 cases. *Lasers Med. Sci.*, **2**, 51–54.

KATO, H., KAWAGUCHI, M., KONAKA, C., NISHIMIYA, K., KAWATE, N., YONEYAMA, K., KINOSHITA, K., NOGUCHI, M., ISHII, M., SHIRAI, M., HIRANO, T., AIZAWA, K. & HAYATA, Y. (1986). Evaluation of photodynamic therapy in gastric cancer. *Lasers Med. Sci.*, **1**, 67–74.

KENNEDY, J.C., POTTIER, R.H. & PROSS, D.C. (1990). Photodynamic therapy with endogenous protoporphyrin IX: basic principles and present clinical experience. *J. Photochem. Photobiol. B: Biol.* **6**, 143–148.

KRASNER, N., CHATLANI, P.T. & BARR, H. (1990). Photodynamic therapy of tumours in Gastroenterology – a review. *Lasers Med. Sci.*, **5**, 233–239.

MALIK, Z. & DJALDETTI, M. (1979). 5-aminolevulinic acid stimulation of porphyrin and hemoglobin synthesis by uninduced Friend erythroleukemic cells. *Cell Differ.*, **8**, 223–233.

MALIK, Z. & LUGACI, H. (1987). Destruction of erythroleukaemic cells by photoactivation of endogenous porphyrins. *Br. J. Cancer*, **56**, 589–595.

MARRIOTT, J. (1968). Regulation of porphyrin synthesis. *Biochem. Soc. Sympo.*, **28**, 61–74.

MILANESI, C., BIOLO, R., REDDI, E. & JORI, G. (1987). Ultrastructural studies on the mechanism of the photodynamic therapy of tumors. *Photochem. Photobiol.*, **46**, 675–681.

MING, S.C., BAJTAI, A., CORREA, P., ELSTER, K., JARVI, O.H., MUNOZ, N., NAGAYO, T. & STEMMERMAN, G.N. (1984). Gastric dysplasia. Significance and pathologic criteria. *Cancer*, **54**, 1794–1801.

NAVONE, N.M., POLO, C.F., FRISARDI, A.L., ANDRADE, N.E. & BATTLE, A.M.D.C. (1990). Heme biosynthesis in human breast cancer – mimetic *in vitro* studies and some heme enzymic activity levels. *Int. J. Biochem.*, **22**, 1407–1411.

NELSON, J.S., LIAW, L.H., ORENSTEIN, A., ROBERTS, W.G. & BERNS, M.W. (1988). Mechanism of tumor destruction following photodynamic therapy with hematoporphyrin derivative, chlorin, and phthalocyanaine. *J. Natl Cancer Inst.*, **80**, 1599–1605.

OFFERHAUS, G.J.A., STADT, J.V., HUIBREGTSE, K. & TYTGAT, G.N.J. (1984). Endoscopic screening for malignancy in the gastric remnant: the clinical significance of dysplasia in gastric mucosa. *J. Clin. Pathol.*, **37**, 748–754.

OFFERHAUS, G.J.A., STADT, J.V., HUIBREGTSE, K., TERSMETTE, A.C. & TYTGAT, G.N.J. (1989). The mucosa of the gastric remnant harbouring malignancy. Histologic findings in the biopsy specimens of 504 asymptomatic patients 15 to 46 years after partial gastrectomy with emphasis on nonmalignant lesions. *Cancer*, **64**, 698–703.

PAQUETTE, B., ALI, H., LANGLOIS, R. & VAN LIER, V.E. (1988). Biological activities of phthalocyanines – VIII. Cellular distribution in V-79 Chinese hamster cells and phototoxicity of selectively sulfonated aluminum phthalocyanines. *Photochem. Photobiol.*, **47**, 215–220.

PATRICE, T., FOULTIER, M.T., YACTAYO, S., ADAM, F., GALMICHE, J.P., DOUET, M.C. & LE BODIC, L. (1990). Endoscopic photodynamic therapy with hematoporphyrin derivative for primary treatment of gastrointestinal neoplasm in inoperable patients. *Dig. Dis. Sci.*, **35**, 545–552.

POPE, A.J. & BOWN, S.G. (1991*a*). The morphological and functional changes in rat bladder following photodynamic therapy with phthalocyanine photosensitization. *J. Urol.*, **145**, 1064–1070.

POPE., A.J., MACROBERT, A.J., PHILLIPS, D. & BOWN, S.G. (1991*b*). The detection of phthalocyanine fluorescence in normal rat bladder wall using sensitive digital imaging microscopy. *Br. J. Cancer*, **64**, 875–879.

POTTER, W.R., MANG, T.S. & DOUGHERTY, T.J. (1987). The theory of photodynamic therapy dosimetry: consequences of photodestruction of sensitizer. *Photochem. Photobiol.*, **46**, 97–101.

RIMINGTON, C. (1966). Porphyrin and heam biosynthesis and its control. *Acta. Med. Scand.*, **179** (Suppl 445), 11–24.

SARDESAI, V.M., WALDMAN, J. & ORTEN, J.M. (1964). A comparative study of porphyrin biosynthesis in different tissues. *Blood*, **24**, 178–186.

SCHMIDT, H.G., RIDDELL, R.H., WALTHER, B., SKINNER, D.B. & RIEMANN, J.F. (1985). Dysplasia in Barrett's esophagus. *J. Cancer Res. Clin. Oncol.*, **110**, 145–152.

SCHRUMPF, E., STADAAS, J., MYREN, J., SERCK-HANSSEN, A., AUNE, S. & OSNES, M. (1977). Mucosal changes in the gastric stump 20–25 years after gastrectomy. *Lancet*, **ii**, 467–469.

SIMA, A.A.F., KENNEDY, J.C., BALKESLEE, D. & ROBERTSON, D.M. (1981). Experimental porphyric neuropathy: a preliminary report. *Can. J. Neurol. Sci.*, **8**, 105–114.

SKINNER, D.B., WALTHER, B.C., RIDDELL, R.H., SCHMIDT, H.G., IASCONE, C. & DEMEESTER, T.R. (1983). Barrett's esophagus. Comparison of benign and malignant cases. *Ann. Surg.*, **198**, 554–565.

TRALAU, C.J., BARR, H., SANDEMAN, D.R., BARTON, T., LEWIN, M.R. & BOWN, S.G. (1987). Aluminum sulfonated phthalocyanine distribution in rodent tumours of the colon, brain, and pancreas. *Photochem. Photobiol.*, **46**, 777–781.

TRALAU, C.J., YOUNG, A.R., WALKER, N.P.J., VERNON, D.I., MACROBERT, A.J., BROWN, S.B. & BOWN, S.G. (1989). Mouse skin photosensitivity with dihaematoporphyrin ether (DHE) and sulphonated phthalocyanine (AlSPc): a comparative study. *Photochem. Photobiol.*, **49**, 305–312.

WEISHAUPT, K.R., GOMER, C.J. & DOUGHERTY, T.J. (1976). Identification of singlet oxygen as the cytotoxic agent in the photoactivation of a murine tumour. *Cancer Res.*, **36**, 2326–2329.

WOLF, P. & KERL, H. (1991). Photodynamic therapy in patients with xeroderma pigmentosum. *Lancet*, **337**, 1613–1614.